# Medical Society of the County of Albany

**Published:** 1876-02

**Authors:** 


ART. III.—*Medical Society of the County of Albany.* Semi-monthly meeting, January 5th, 1876.

Dr. Henry March, President, in the Chair.

Dr. McFalls, of St. Lawrence county, was introduced to the society, and invited to participate in the proceedings.

Dr. Frederic C. Curtis read a very interesting paper on *Certain Points Connected with Typhoid Fever.*—See Art. II (this Journal).

Dr. Beckett enquired how many more cases of typhoid fever had been noticed since the introduction of the river water. He said that it was noticed as early as last spring that typhoid fever was very prevalent, and it was laid to the river water. But this is not true, for river water was not introduced then. Typhoid fever may be caused from other sources. Sand creek, which gave the main supply before, runs by the cattle-yards at West Albany, the highways, etc., and I have often seen numbers of cattle drinking from it. Dr. Curtis stated that typhoid fever had not been more frequent in poor families than in rich familes. Lately, in the alteration of some houses iu England, some of the water pipes were found to have been left open leading to the water-closet, and when the water was drawn off emanations from the closet arose. It may be the same here. Our water has been better since the river has been brought in the city than before. Before we say it is the cause of typhoid fever we need to try it longer.

Dr. Benjamin enquired what was the usual range of temperature in typhoid fever.

Dr. Curtis replied that the temperature does not range very high. It is more important to observe the relation of the morning to the evening temperature.

Dr. Benjamin said his reason for asking was that in the recent case of Swartz vs. the Orphan Asylum, where disease was said to be caused by the emanations from the slaughter-house, Dr. E. R. Hun said it was not typhoid because the temperature ranged from 102° to 103°; but he did not agree with the doctor, and was supported in his opinion by Dr. Swinburne. As to the river water, the doctor said he had an extended range of observation, but had had only one case of typhoid fever since its introduction.

Dr. Curtis said in regard to the temperature, 103° was high. From 102° to 103° is generally found to be the range.

Dr. McFalls asked if any gentleman present had had any experience with cold-water baths in typhoid fever or typhoid pneumonia.

Dr. James S. Bailey said in his early years of practice in Alabama, an epidemic of typhoid fever was prevailing malignantly on some of the plantations, and a physician went to a woman who had several negroes very ill with it and said if she would change her physician and employ him, he would have her negroes in the field in two weeks. She employed him, and he kept his word, and in the specified time the negroes were in the field, but under the ground. The doctor used sheets wet with cold water.

Dr. MERENESS said that Dr. McFalls had related to him a very interesting case, on their way to the meeting, and he would like to hear the doctor repeat it.

Dr. McFALLS said the case recently occurred in his practice. It was a case of typhoid pneumonia which had prevailed in that section of the county. He was invited to see the son of a neighboring physician who was very ill. He found him in a critical condition, with low muttering delirium, temperature 103°, tongue dry and could not be protruded. As the case was so desperate and did not respond to treatment, Dr. McFalls proposed the cold bath. Wet sheets were applied instead of the cold bath. He also gave 10 grs. of quinine, then increased to 15 grs., and finally as high 25 grs., at one dose. The next morning, before leaving his patient, he ordered the treatment continued until his return the next day. Fearing the father might be afraid to use so large doses of quinine, the doctor, on his arrival home, sent a young physician to keep up the treatment. He returned on the morrow, and he found the patient doing well—temperature 102°. This, however, increased, and he gave 25 gains of qninine, which had the effect to reduce the temperature again. The right lung was solid almost throughout, but there was no cough. The case finally made a good recovery.

Dr. VAN DERVEER referred to the different modes of applying cold water, viz.: wet sheet, plunge bath, and, as Thompson recommends, by a cold-water bed. Spencer Wells uses after his operations for ovariotomy an ice-water cap, consisting of a simple rubber bag in different compartments which fit the head only. By a coil a stream of water was passed through the cap which had the desired effect. He thought it would answer well in cases of this sort.

Dr. MOSCHER said if he could find patients who would submit to the application of cold water, he would use it very generally, but he thought in this country the people are not educated in the use of cold water. It is a serious thing to use it in the treatment of scarlet fever. It is an important thing to reduce the temperature, and he favored the use of cold water.

Dr. COOK asked if in the use of cold water stimulants were not also used largely.

Dr. VAN DERVEER presented a specimen of meningial apoplexy. The case was one of Dr. Papens', which had the following history. He was called to attend a case of confinement, the patient being young, aged 19, promipara. On examination, found the head presenting; remained a little while, and left with the understanding that should anything occur during the night to send for him. The next morning he was summoned and found the patient in a comatose condition. She was taken in a fit about four hours before his arrival. In about half an hour she had five or six convulsions. He saw her again in three hours more; her con. dition was unchanged, though not convulsed so often. She had been taking hydrate chloral; when taking the last dose she did not swallow. Three hours later the convulsions had increased. Treatment was suspended. Death took place soon after.

The specimen showed a clot in the subarachnoid sac around the medulla, and completely filling the ventricles. In Dr. Hammond's report of Vice-President Wilson's case he laid great stress on the clot around the medulla. A clot there will cause instant death. Dr. Beckett had had a similar case which he was asked to relate.

Dr. BECKETT said that the patient had had the day previous to his death a severe headache and continued until the next morning. About noon he asked for some beef tea, and arose form the bed and walked into an adjoining room and sat down. At that time he attempted to speak, fell over and died. At the autopsy a clot was found in the meninges, and all around the medulla.

Dr. VAN DERVEER then presented another specimen which was also from a patient treated by Dr. Beckett who related the history of this case also.

Dr. BECKETT said on the 20th of March last, the patient was taken with vomiting blood. Had several hemorrhages between that time and the 24th. From that time until June was pretty well, but then had another attack when riding in the buggy. He continued more or less ill until December when the hemorrhage destroyed his life.

Dr. VAN DERVEER reported the autopsy. John F., æt. 46, butcher, body well nourished, several depressed white cicatrices were found on the lower limbs, and around the knees, also cicatrices on the glans penis. Body was opened by conical incision. In making the longitudinal incision a small cyst was found near the umbilicus, but no connection could be found between it and the abdominal cavity. No fluid in the abdominal cavity.

*Thorax—left lung—* Some slight adhesions were found at the lower part of the upper lobe, and also at the posterior part of the lower lobe. On section the bronchial tubes were found much congested with considerable œdema.

*Right lung*—Adhesions at the upper lobe. Same condition also present as in left; both crepitated.

*Heart*—Slight fibrinous clots in left ventricle atheromatous deposits on aorta and ulcerations on the inner coats, supposed to be syphilitic, walls of heart soft and tear easily. Weight without pericardium 1½ lbs.; valves normal.

*Abdominal cavity—Liver*; firm adhesions of the upper surface of the upper lobe to the diaphragm. Adhesions of the under surface of the lower lobe to the ascending colon at its junction with the transverse colon. The organ was contracted—edges rounded, "hob nailed" and very pale in color. On section the granular condition could be seen.

*Spleen* greatly enlarged, weighed 4½ lbs. Long diameter, ten inches; short diameter, 6½ inches; thickness, 3 inches.

*Kidneys*—(left) Supra-renal capsule normal, kidneys large and waxy. The "large white" kidney. Capsule slightly adherent—long diameter, 6 inches; short diameter, 3 inches. Right same as left.

*Intestines*—Vermiform appendix normal. Four inches from ileocoecal valve, small intestines very much discolored for the distance of 8 feet. Partially filled with blood.

Syphilitic cicatrices were found in the rectum.

*Pancreas* normal.

*Stomach* contained about three pints of fluid blood. No rupture of any blood vessel could be found, but mucous membrane was softened and congested.

*Spine*—A lateral curvature was found existing at the 1st and 2d lumbar verbetræ which were twisted to the right on the third. The sacrum pointed in a line drawn from the right ear to the symphysis pubis. The variation of the column was 3-4 of an inch to the right of the median line. Dorsal vertebræ inclined slightly towards the left. An exostosis was found projecting about 3-4 of an inch from the right side of the body of the seventh dorsal vertebræ.

Adjourned.

